# Real-world data shows increased reactogenicity in adults after heterologous compared to homologous prime-boost COVID-19 vaccination, March−June 2021, England

**DOI:** 10.2807/1560-7917.ES.2021.26.28.2100634

**Published:** 2021-07-15

**Authors:** Annabel A Powell, Linda Power, Samantha Westrop, Kelsey McOwat, Helen Campbell, Ruth Simmons, Mary E Ramsay, Kevin Brown, Shamez N Ladhani, Gayatri Amirthalingam

**Affiliations:** 1 Immunisation and Countermeasures Division, Public Health England, London, United Kingdom; 2 London School of Hygiene and Tropical Medicine, London, United Kingdom; 3 Paediatric Infectious Diseases Research Group, St. George’s University of London, London, United Kingdom

**Keywords:** Reactogenicity, heterologous schedule, COVID-19 vaccine, Comirnaty, Vaxevria, prime-boost, BNT162b2, ChAdOx1/nCoV-19

## Abstract

Adults receiving heterologous COVID-19 immunisation with mRNA (Comirnaty) or adenoviral-vector (Vaxzevria) vaccines had higher reactogenicity rates and sought medical attention more often after two doses than homologous schedules. Reactogenicity was higher among ≤ 50 than > 50 year-olds, women and those with prior symptomatic/confirmed COVID-19. Adults receiving heterologous schedules on clinical advice after severe first-dose reactions had lower reactogenicity after dose 2 following Vaxzevria/Comirnaty (93.4%; 95% confidence interval: 90.5–98.1 vs 48% (41.0–57.7) but not Comirnaty/Vaxzevria (91.7%; (77.5–98.2 vs 75.0% (57.8–87.9).

Concerns about vaccine-induced thrombosis and thrombocytopenia syndrome (VITTs) following vaccination with (coronavirus disease) COVID-19 adenoviral vector vaccines has led to several countries recommending an mRNA vaccine for the second dose in younger adults who had been given an adenoviral vector vaccine for their first dose [1-4]. A clinical trial in England (COM-COV) reported that heterologous schedules using mRNA-based Comirnaty (BNT162b2, BioNTech-Pfizer, Mainz, Germany/New York, United States) hereafter referred to as BNT, and adenovirus vector Vaxrevia (ChAdOx1/nCoV-19, AstraZeneca, Cambridge, United Kingdom), hereafter ChAd, COVID-19 vaccines after a 4-week interval were associated with increased reactogenicity after the booster dose compared with their homologous counterparts, although none required hospitalisation [5].

Here we report on the real-world effects of heterologous prime-boost COVID-19 vaccination following the UK extended schedule of up to 12 weeks between doses.

## Recruitment and participation

**Figure 1:**
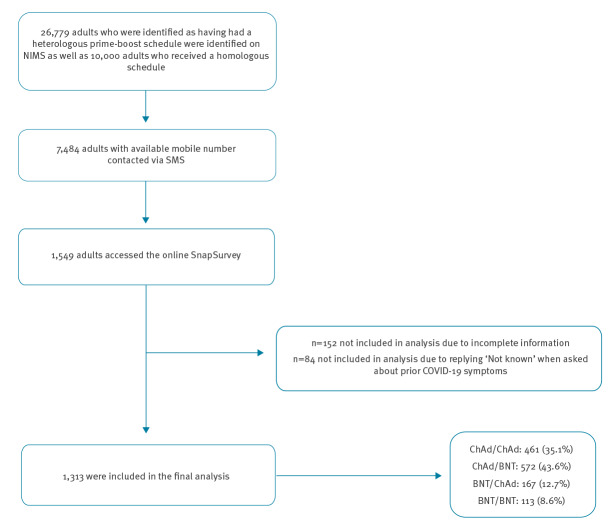
Flow diagram of recruitment and participation of individuals into study on reactogenicity in adults after heterologous compared to homologous prime-boost COVID-19 vaccination, 29 March−1 June 2021, England

BNT: Comirnaty (BNT162b2, BioNTech-Pfizer, Mainz, Germany/New York, United States); ChAd: Vaxrevia (ChAdOx1/nCoV-19, AstraZeneca, Cambridge, United Kingdom); NIMS: National Immunisation Management System.

We used the National Immunisation Management System (NIMS) database to identify adults aged 18–75 years recorded as having received a heterologous prime-boost schedule in England. We identified 26,779 adults with a heterologous schedule between 29 March 2021 and 01 June 2021, initially in London, the South East and East of England but later extended nationally. In addition, 10,000 adults who received a homologous prime-boost schedule on 01 June 2021 in England were identified through NIMS on 08 June 2021 [6]. Of these, 7,484 individuals who were recorded as receiving their second dose in the previous 21 days and had provided a mobile phone number were texted a link to an online survey using SnapSurvey. 1,549 individuals accessed the online survey, 1,397 completed the questionnaire and 1,313 were included in the analysis which gave a response rate of 18.7% (Figure 1). Statistical analysis was performed using STATA/SE v.15.1 and graphs created in RStudio [7].

The median age of those who had ChAd/BNT was 49 (interquartile range (IQR): 38–59) years, 76.4% (437/572) were women and 90.0% (515/572) were white; for BNT/ChAd this was 56 (IQR: 44–64), 64.7% (108/167) and 88.6% (148/167) respectively. This compared with 52 (IQR: 46–56) years, 61.2% (282/461) women and 86.6% (399/461) white for participants receiving ChAd/ChAd and 51 (IQR: 34–60), 71.7 (81/113) and 77.9% (88/113), respectively, for BNT/BNT recipients (Supplementary material 2). A higher proportion of participants had ChAd than BNT as their first dose because of vaccine supply in England at the time (Supplementary material 2).

## Heterologous vs homologous schedules

The median time between doses for homologous vs heterologous schedules was 69 (range: 65–77) days and 76 (range: 67–85) days, respectively. Among previously uninfected participants (886/1,313; 67.5%), ChAd/ChAd recipients had significantly higher reactogenicity following their first dose than second dose (63.8%; 95% confidence interval (CI): 57.9−69.3 vs 33.5%; 95% CI: 28.0−39.2), while BNT/BNT recipients had similar reactogenicity following both doses (33.3%; 95% CI: 23.4−44.5 vs 33.3%; 95% CI: 23.4−44.5) (Supplementary material 3). Having prior COVID-19 symptoms (300/1,313; 22.8%) or confirmed COVID-19 (127/1,313; 9.7%) was generally associated with higher reactogenicity after the first dose for both vaccine brands, but particularly among those receiving BNT first. (Supplementary material 3 and 6). (Table 1).

**Table 1:** Number of participants who reported local and systemic symptoms after prime and boost vaccines by vaccination schedule, study on reactogenicity in adults after heterologous compared to homologous prime-boost COVID-19 vaccination, 29 March−1 June 2021, England

Reactions	Priming vaccine dose								Booster vaccine dose							
	ChAd/ChAd (n =461)		ChAd/BNT (n = 572)		BNT/ChAd (n = 167)		BNT/BNT (n = 113)		ChAd/ChAd (n = 461)		ChAd/BNT (n = 572)		BNT/ChAd (n = 167)		BNT/BNT (n = 113)	
	n	% (95%CI)	n	% (95%CI)	n	% (95%CI)	n	% (95%CI)	n	% (95%CI)	n	% (95%CI)	n	% (95%CI)	n	% (95%CI)
Systemic																
Fever	188	40.9 (36.3–45.5)	316	55.2 (51.0–59.4)	30	18.0 (12.5–24.6)	13	9.8 (5.3–16.1)	29	6.3 (4.3–8.9)	93	16.3 (13.3–19.5)	46	27.5 (20.9–35.0)	12	9.0 (4.7–15.2)
Chills	196	42.6 (38.0–47.2)	296	51.7 (47.6–55.9)	21	12.6 (8.0–18.6)	15	11.3(6.5–17.9)	40	8.7 (6.3–11.7)	108	18.9 (15.8–22.3)	46	27.5 (20.9–35.0)	12	9.0 (4.7–15.2)
Headache	200	43.5 (38.9–48.1)	370	64.7 (60.6–68.6)	37	22.2 (16.1–29.2)	24	18.0 (11.9–25.6)	93	20.2 (16.6–24.2)	195	34.1 (30.2–38.1)	70	41.9 (34.3–50.0)	18	13.5 (8.2–20.5)
Unwell	244	53.0 (48.4–57.7)	405	70.8 (66.9–74.5)	49	29.3 (22.6–36.9)	28	21.1 (12.5–28.9)	84	18.3 (14.8–22.1)	199	34.8 (30.9–37.9)	70	41.9 (34.3–50.0)	22	16.5 (10.7–24.0)
Tiredness	241	52.4 (47.7–57.0)	403	70.5 (66.5–74.1)	47	28.1 (21.5–35.6)	28	21.1 (12.5–28.9)	106	23 (19.3–27.2)	221	38.6 (34.6–42.8)	72	43.1 (35.5–51.0)	26	19.5 (13.2–27.3)
Joint pain	184	40 (35.5–44.6)	298	52.1 (47.9–56.3)	27	16.2 (10.9–22.6)	17	12.8 (7.6–19.7)	59	12.8 (9.9–16.2)	130	22.7 (19.4–26.4)	50	29.9 (23.1–37.5)	13	9.8 (5.3–16.1)
Nausea	49	10.7 (8.0–13.8)	153	26.7 (23.1–30.6)	20	12.0 (7.5–17.9)	4	3.0 (0.8–7.5)	14	3.0 (1.7–5.1)	58	10.1 (7.8–12.9)	21	12.6 (8.0–18.6)	3	2.3 (4.7–6.5)
Local																
Pain	203	44.1 (39.5–48.8)	306	53.5 (49.3–57.6)	49	29.3 (22.6–36.9)	35	26.3 (19.1–34.7)	88	19.1 (15.6–23.0)	235	41.1 (37.0–45.2)	64	38.3 (30.9–46.2)	24	18.0 (11.9–25.6)
Tenderness	197	42.8 (38.3–47.5)	292	51 (46.8–55.2)	44	26.3 (19.8–33.7)	37	27.8 (20.4–36.3)	89	19.3 (15.8–23.3)	253	44.2 (40.1–48.4)	65	38.9 (31.5–46.8)	28	21.1 (12.5–28.9)
Itch	27	5.9 (3.9–8.4)	41	7.2 (5.2–9.6)	13	7.8 (4.2–12.9)	3	2.3 (4.7–6.5)	14	3.0 (1.7–5.1)	25	4.4 (28.5)	7	4.2 (1.7–8.4)	3	2.3 (4.7–6.5)
Redness	67	14.6 (11.5–18.1)	117	20.5 (17.2–24.0)	19	11.4 (7.0–17.2)	10	7.5 (3.7–13.4)	23	5.0 (3.2–7.4)	71	12.4 (9.8–15.4)	21	12.6 (8.0–18.6)	5	3.8 (1.2–8.6)
Total																
Systemic	303	65.9 (61.3–70.2)	460	80.4 (76.9–83.6)	66	39.5 (32.0–47.4)	38	28.6 (21.1–37.0)	147	32 (27.7–36.4)	292	51 (46.9–55.2)	91	54.5 (46.6–62.2)	35	26.3 (19.1–34.7)
Local	246	53.5 (48.8–58.1)	349	61(56.9–65.0)	61	36.5 (29.2–44.3)	42	31.6 (23.8–40.2)	119	25.9 (21.9–30.1)	297	51.9 (47.7–56.1)	74	44.3 (36.6–52.2)	34	25.6 (18.4–33.8)
Overall	307	66.7 (62.2–71.0)	474	82.7(79.5–85.9)	80	47.9 (40.1–55.8)	46	34.6 (26.6–43.3)	162	35.2 (30.8–39.8)	346	60.5 (56.4–64.5)	98	58.8 (50.8–66.2)	39	29.3 (21.8–37.8)
Medical attention	34	7.4 (5.2–10.2)	188	32.9 (29.0–36.9)	35	21.0 (15.1–27.9)	8	7.1 (3.1–13.5)	13	2.8 (1.5-4.7)	55	9.6 (7.3-12.3)	31	18.8 (13.0-25.3)	7	6.2 (2.1–10.5)

BNT: Comirnaty (BNT162b2, BioNTech-Pfizer, Mainz, Germany/New York, United States); ChAd: Vaxrevia (ChAdOx1/nCoV-19, AstraZeneca, Cambridge, United Kingdom).

Please note individuals could report more than one reaction therefore totals do not match the sum of the symptoms.

After the second vaccine dose, previously uninfected adults in both heterologous vaccination groups had significantly higher reactogenicity than their homologous counterparts, with similar rates among those receiving ChAd/BNT (54.4%; 95% CI: 49.4–59.5) and BNT/ChAd (55.2%; 95% CI: 46.1–64.1) compared with ChAd/ChAd (33.5%; 95% CI: 28.0–39.2) or BNT/BNT (33.3%; 95% CI: 23.4–44.5). Similar trends were observed among previously symptomatic and confirmed COVID-19 cohorts (Supplementary material 3 and 6).

## Age and sex

Reactogenicity was generally higher in ≤ 50 year-olds compared with > 50 year-olds for both doses across immunisation schedules, including after the second dose in those receiving heterologous compared with homologous schedules, although only significant for BNT/ChAd 76.3% (95% CI: 59.8–88.6) in ≤ 50 year-olds vs 46.0% (95% CI: 35.2–57.0) in > 50 year-olds (Supplementary material 4 and 6). Reactogenicity was higher in women than men for both doses across immunisation schedules (Supplementary material 5 and 6).

## Schedule change because of severe reactogenicity

The most common reason for receiving a heterologous schedule was following clinical advice because of severe reactogenicity after the first dose (290/739, 39.2%). In this cohort, reactogenicity after the second dose was significantly lower than the first dose for ChAd/BNT (93.4%; 95% CI: 90.5–98.1 vs 48%; 95% CI: 1.0–57.7) but not BNT/ChAd (91.7%; 95% CI: 77.5–98.2 vs 75.0%; 95% CI: 57.8–87.9). (Table 2). 

**Table 2:** Number of participants who completed a heterologous schedule following clinical advise after a severe reaction to the first dose, with no previous symptoms of COVID-19 or confirmed infection who reported local and systemic symptoms after prime and boosting doses of COVID-19 vaccines by vaccination schedule, 29 March−1 June 2021, England

Reactions	Prime				Boost			
	ChAd/BNT (n=152)		BNT/ChAd (n = 36)		ChAd/BNT (n = 152)		BNT/ChAd (n =36)	
	n	% (95%CI)	n	% (95%CI)	n	% (95%CI)	n	%(95%CI)
Systemic								
Fever	90	59.2 (52.6–68.7)	13	36.1 (20.8–53.8)	16	10.8 (6.3–17.0)	11	30.6 (16.3–48.1)
Chills	88	57.9 (51.1–67.4)	13	36.1 (20.8–53.8)	29	19.1 (13.5–26.9)	17	47.2 (30.4–64.5)
Headache	105	69.1 (61.1–76.3)	16	44.4 (28.0–61.9)	42	27.6 (21.3–36.4)	19	52.8 (35.5–69.6)
Unwell	115	75.6 (70.1–84.1)	21	58.3 (40.8–74.5)	38	25 (18.9–33.5)	17	47.2 (30.4–64.5)
Tiredness	108	71.1 (65.1–79.9)	20	55.6 (38.1–72.1)	39	25.7 (19.5–34.2)	20	55.6 (38.1–72.1)
Joint pain	86	56.6 (49.7–66.2)	15	41.7 (25.5–59.2)	20	13.2 (8.5–20.1)	14	38.9 (23.1–56.5)
Nausea	58	38.2 (31.3–47.5)	10	28.0 (14.2–45.2)	12	7.9 (4.3–13.7)	5	13.9 (4.7–29.5)
Local								
Pain	80	52.6 (45.7–62.3)	21	58.3 (40.8–74.5)	46	30.3 (23.7–39.2)	16	44.4 (28.0–61.9)
Tenderness	69	45.4 (38.4–55.0)	20	55.6 (38.1–72.1)	43	28.3 (21.9–37.1)	17	47.2 (30.4–64.5)
Itch	17	11.2 (6.8–17.6)	5	13.9 (4.7–29.5)	8	5.3 (2.4–10.4)	1	2.8 (0.1–14.5)
Redness	32	21.1 (83.8–94.2)	6	16.7 (6.4–32.8)	11	7.2 (3.8–12.9)	6	16.7 (6.4–32.8)
Total								
Systemic	133	87.5 (52.5–68.7)	24	66.7 (49.0–81.4)	63	41.5 (34.5–50.1)	25	69.4 (51.9–83.7)
Local	90	59.2 (90.5–98.1)	26	72.2 (54.8–85.8)	57	37.5 (30.6–46.9)	19	52.8 (35.5–69.6)
Overall	141	93.4 (90.5–98.1)	33	91.7 (77.5–98.2)	73	48.0 (41.0–57.7)	27	75.0 (57.8–87.9)
Medical attention	93	61.2 (53.0–69.0)	17	47.2 (30.4–64.5)	10	6.6 (3.2-11.8))	7	19.4 (8.2–36.0)

BNT: Comirnaty (BNT162b2, BioNTech-Pfizer, Mainz, Germany/New York, United States); ChAd: Vaxrevia (ChAdOx1/nCoV-19, AstraZeneca, Cambridge, United Kingdom).

Please note individuals could report more than one reaction therefore totals do not match the sum of the symptoms.

Other reasons for receiving a heterologous schedule included supply issues, individuals requesting a different vaccine, receiving a different vaccine by mistake or other reasons including family history of clotting or recently pregnant. In this cohort, too, reactogenicity was higher in those receiving a heterologous compared with homologous schedules (Table 3).

**Table 3:** Number of participants who completed a homologous schedule and those who completed a heterologous schedule following clinical advise despite not having had a severe reaction to the prime dose, with no previous symptoms of COVID-19 or confirmed infection who reported local and systemic symptoms after prime and boosting doses of COVID-19 vaccines by vaccination schedule, March-May 2021, England

Reactions	Prime								Boost							
	ChAd/ChAd (n=287)		ChAd/BNT (n = 238)		BNT/ChAd (n = 89)		BNT/BNT (n = 84)		ChAd/ChAd (n = 287)		ChAd/BNT (n = 238)		BNT/ChAd (n = 89)		BNT/BNT (n = 84)	
	n	% (95%CI)	n	% (95%CI)	n	% (95%CI)	n	% (95%CI)	n	% (95%CI)	n	% (95%CI)	n	% (95%CI)	n	% (95%CI)
Systemic																
Fever	104	36.2 (30.6–42.0)	105	44.1 (37.7–50.7)	3	3.4 (0.7–9.5)	6	7.1 (3.7–14.9)	17	5.9 (3.5–9.3)	36	15.1 (10.8–20.3)	23	25.8 (17.1–36.2)	6	7.1 (2.7–14.9)
Chills	119	41.5 (35.7–47.4)	109	45.8 (39.4–52.4)	0	0 (0–4.0)	6	7.1 (3.7–14.9)	24	8.4 (5.4–12.2)	43	18.1 (13.4–23.6)	19	23.4 (13.4–31.3)	7	8.3 (3.4–16.4)
Headache	109	38.0 (32.3–43.9)	132	55.5 (48.9–61.9)	6	6.7 (2.5–14.1)	12	14.3 (7.6–23.6)	48	16.7 (12.6–21.6)	77	32.4 (26.5–38.7)	25	28.1 (19.1–38.6)	12	14.3 (7.6–23.6)
Unwell	141	49.1 (43.2–55.1)	144	60.5 (54.0–66.8)	8	9.0 (4.0–17.0)	13	15.5 (8.5–25.0)	53	18.5 (14.1–23.4)	81	34.0 (28.0–40.4)	31	34.8 (25.0–45.7)	15	17.9 (10.4–27.7)
Tiredness	138	48.1 (42.2–54.0)	146	61.3 (54.8–67.6)	9	10.1 (4.7–18.3)	15	17.7 (10.4–27.7)	60	20.9 (16.4–26.1)	90	37.8 (31.6–44.3)	27	30.3 (21.0–41.0)	19	22.6 (14.2–33.0)
Joint pain	105	36.6 (31.0–42.5)	104	43.7 (37.3–50.3)	5	5.6 (1.9–12.6)	7	8.3 (3.4–16.4)	37	12.9 (9.2–17.3)	54	22.7 (17.5–28.5)	22	24.7 (16.2–35.0)	9	10.7 (5.0–19.4)
Nausea	27	9.4 (6.3–13.4)	41	17.2 (12.7–22.6)	2	2.3 (0.3–7.9)	1	1.2 (0.0–6.5)	5	1.7 (0.6–4.0)	22	9.2 (5.9–13.7)	5	5.6 (1.9–12.6)	1	1.2 (0.0–6.5)
Local																
Pain	117	40.8 (35.0–46.7)	119	50.0 (43.5–56.5)	9	9.0 (4.0–17.0)	21	25.0 (16.2–38.2)	57	19.9 (15.4–25.0)	101	41.4 (36.1–49.0)	24	27.0 (18.1–37.4)	17	20.2 (12.3–30.4)
Tenderness	113	39.4 (33.7–45.3)	115	48.3 (41.8–54.9)	7	7.9 (3.2–15.5)	23	27.4 (18.2–38.2)	57	19.9 (15.4–25.0)	106	44.5 (38.1–51.1)	25	28.1 (19.1–38.6)	20	23.8 (15.2–34.3)
Itch	13	4.5 (2.4–7.6)	8	3.4 (1.5–6.5)	2	2.3 (0.3–7.9)	1	1.2 (0.0–6.5)	9	3.1 (1.4–5.90)	7	2.9 (1.2–6.0)	4	4.5 (1.2–11.1)	1	1.2 (0.0–6.5)
Redness	35	12.2 (8.6–16.6)	39	16.4 (11.9–21.7)	5	5.6 (1.9–12.6)	4	4.8 (1.3–11.7)	10	3.5 (1.7–6.3)	27	11.3 (7.6–16.1)	8	10.1 (4.7–18.3)	1	1.2 ( 0.0–6.5)
Total																
Systemic	180	62.7 (56.8–68.3)	167	70.2 (63.9–75.9)	17	19.1 (11.5–28.8)	21	25.0 (16.2–38.2)	82	28.6 (23.4–34.2)	114	47.9 (41.4–54.5)	37	41.6 (31.2–52.5)	24	28.6 (19.2–39.5)
Local	142	49.5 (43.6–55.4)	137	57.6 (51.0–63.9)	12	13.5 (7.2–22.4)	25	29.8 (20.3–40.7)	73	25.4 (20.5–30.9)	122	51.3 (44.7–57.8)	30	33.7 (24.0–44.5)	25	29.8 (20.3–40.7)
Overall	183	63.8 (57.9–69.3)	172	72.3 (66.1–77.9)	20	22.5 (12.3–32.6)	28	33.3 (23.4–44.5)	96	33.5 (28.0–39.2)	139	58.4 (51.9–64.7)	42	47.2 (36.5–58.1)	28	33.3 (23.4–44.5)
Medical attention	19	6.6 (4.0–10.2)	34	14.3 (10.1–19.4)	5	5.6 (1.9–12.6)	4	4.8 (1.3–11.8)	8	2.8 (1.2-5.4)	18	7.6 (4.5–11.7))	11	12.4 (6.3-21.0)	5	6.0 (2.0–13.4)

BNT: Comirnaty (BNT162b2, BioNTech-Pfizer, Mainz, Germany/New York, United States); ChAd: Vaxrevia (ChAdOx1/nCoV-19, AstraZeneca, Cambridge, United Kingdom).

Please note individuals could report more than one reaction therefore totals do not match the sum of the symptoms.

## Severity of reactions

In this real-world setting, 20.1% (265/1,313) participants required medical attention i.e. emergency department or hospitalisation, after their first dose; 32 for severe allergic reaction (1 ChAd/ChAd, 18 ChAd/BNT, 12 BNT/ChAd, 1 BNT/BNT) including 10 with anaphylaxis, 28 because of clotting events (ChAd/BNT) and 205 for other reasons including dizziness, fever, rash, dyspnoea, limb swelling, chest pain, loss of vision, abdominal pain and nausea. Most participants with symptoms following either dose of vaccine, reported onset within 48 hours post vaccination.

After the second dose, 8.1% (106/1,313) individuals overall required medical attention, including 55 who reported requiring medical attention after the first dose, with a higher proportion after a heterologous schedule. This was significant for ChAd/BNT (9.6%; 95% CI: 7.3-12.3) compared with ChAd/ChAd (2.8%; 1.5–4.7), as well as BNT/ChAd (18.6%; 95% CI: 13.0-25.3) compared with BNT/BNT (6.2%; 95% CI: 2.1–10.5) (Table 1). Of those who received a heterologous schedule because of severe reactogenicity after the first dose, observations were similar, as were observations for those receiving heterologous schedules for other reasons, albeit non-significant. 

**Figure 2:**
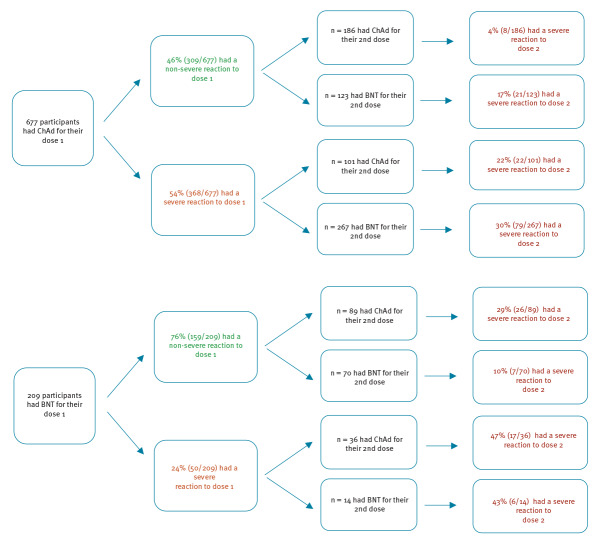
Flow diagram of participants with neither previous symptoms of COVID-19 nor confirmed infection who reported severe reactions (Grades 3 or 4) for their second dose of the vaccine by those that reported severe reactions after their first dose and those that reported non-severe reactions (Grades 0–2) after their first dose by vaccination schedule

BNT: Comirnaty (BNT162b2, BioNTech-Pfizer, Mainz, Germany/New York, United States); ChAd: Vaxrevia (ChAdOx1/nCoV-19, AstraZeneca, Cambridge, United Kingdom).

A high proportion (29.7%, 124/418) of individuals with severe reaction after dose 1 reported a severe reaction again to dose 2, irrespective of their vaccine schedule; this proportion was highest for BNT/ChAd. In those who reporting no, mild or moderate reactions following dose 1, both heterologous cohorts were more likely to report severe symptoms following dose 2: ChAd/BNT (17.1%; 95% CI: 10.9–24.9) vs ChAd/ChAd (4.3%; 95% CI: 1.9–8.3) and BNT/ChAd (29.1%; 95% CI: 20.1–39.8) vs BNT/BNT (10.0%; 95% CI: 4.1–19.5) (Figure 2, Supplementary material 7).

Grade 0 = No symptoms, Grade 1 = Mild – easily tolerated with no limitation on normal activity, Grade 2 = Moderate – some limitation of daily activity, Grade 3 = Severe – unable to perform normal daily activity and Grade 4 = Emergency department or hospital admission required [5].

## Ethical approval

Public Health England has legal permission, provided by Regulation 3 of The Health Service (Control of Patient Information) Regulations 2002, to process patient confidential information for national surveillance of communicable diseases and as such, individual patient consent is not required to access records. Individual patient consent was obtained by those who completed the questionnaire.

## Discussion

COVID-19 vaccines have been deployed at scale with great success in many countries, but most of the global population remains unvaccinated [8]. In Europe, most vaccinated individuals have received mRNA or adenoviral-vector vaccines. For two-dose schedules, individuals are recommended to receive the same vaccine brand because of a lack of data on heterologous schedules. For some, however, it may be necessary to offer a different type of vaccine for their second dose if, for example, they experienced severe anaphylaxis after their first dose. Given the global demand for COVID-19 vaccines and potential interruptions in supply, vaccine shortages may lead to policies recommending a heterologous vaccine schedule to provide more rapid protection, especially in the context of new variants, where one dose may provide only partial protection [9].

Following national implementation of COVID-19 vaccines in England, we found that previously-uninfected individuals who received heterologous prime-boost schedules were 2.4 times (27.8% vs. 11.6%) more likely to report severe reactogenicity, including increased requirement for medical attention, after their second dose than those receiving homologous schedules. These findings were irrespective of the reason for receiving a heterologous schedule. Reactogenicity rates were higher in younger adults, women and after the first dose of ChAd in any schedule. Those experiencing severe reactions after their first dose, irrespective of the vaccine type, were more than twice as likely to experience a severe reaction after the second dose compared to those reporting a no or a mild-to-moderate reaction after their first dose (29.7% vs. 13.3%). Our findings suggest that adults who have a severe reaction to their first dose should be advised that their risk of a severe reaction after their second dose will be higher with a heterologous schedule than a homologous schedule. Therefore, completion with the same vaccine brand should be considered unless there is clear evidence of anaphylaxis or other contraindications such as VITTs.

Our study is consistent with existing trial data reporting increased reactogenicity after ChAd prime but not with data reporting increased reactogenicity after BNT/BNT boost [10,11]. It is also consistent with the COM-COV study reporting increased reactogenicity following either heterologous schedule using a 4-week interval in healthy individuals [5]. Our real-world data in individuals receiving the UK-recommended 8–12 week extended schedule included a higher proportion of women, under 50 year-olds and at least 10% of participants with prior COVID-19. All these factors may have contributed to the higher proportion of people reporting severe reactions or requiring medical attention. This contrasts with multiple studies in Germany that reported little difference in reactogenicity between homologous and heterologous schedules [12-14]. While these studies were based on similar extended schedules for heterologous doses, none included a BNT/ChAd schedule. One study compared reactogenicity after ChAd/BNT with both homologous schedules [12], while others used BNT/BNT for comparison [13,14]. A Spanish clinical trial also reported mild reactogenicity overall following an extended ChAd/BNT schedule [15], but did not have a control arm, instead comparing reactogenicity to non-contemporaneous clinical trial data of homologous ChAd and BNT schedules [10,11]. Choice of controls, demographics including age, sex and prior infection may account for some of the observed differences.

The strength of our study is the use of real-world data to assess reactogenicity and need for medical attention after different COVID-19 vaccines and schedules. Our findings are, however, only applicable to heterologous immunisation with mRNA and adenoviral vector vaccines. Other potential limitations include recruitment bias towards those with more severe reactions and recall bias because participants completed the questionnaire after their second dose. We also relied on participants reporting COVID-19 symptoms and diagnosis before vaccination. These potential biases would, however, have been equivalent across the different schedules.

Emerging immunogenicity studies indicate robust immune responses after heterologous immunisation in animal models [16], and increased antibody titres, cellular responses and neutralising activity against variants-of-concern in adults receiving ChAd/BNT [12-14]. If confirmed in further studies, the benefits of better and potentially longer protection following heterologous schedules will need to be carefully assessed against increased reactogenicity as we have reported here. These findings will also have implications for considerations on the needs of future boosting doses.

## Supplementary Data

750000 Supplementary Material

## References

[1] Swedish Public Health Agency. Recommendation for a 65-year age limit for AstraZeneca's vaccine remains. [Swedish]. Stockholm: Folkhälsomyndigheten; 2021. Available from: https://www.folkhalsomyndigheten.se/nyheter-och-press/nyhetsarkiv/2021/april/rekommendation-om-aldersgrans-pa-65-ar-for-astrazenecas-vaccin-kvarstar/

[2] Haute Autorité de Santé. Covid-19: what vaccine strategy for people under 55 who have already received a dose of AstraZeneca? [French]. Saint-Denis: Haute Autorité de Santé; 2021. Available from: https://www.has-sante.fr/jcms/p_3260335/

[3] Danish Health Authority. Denmark continues its vaccine rollout without the COVID-19 vaccine from AstraZeneca. Copenhagen: Danish Health Authority; 2021. Available from: https://www.sst.dk/en/English/news/2021/Denmark-continues-its-vaccine-rollout-without-the-COVID-19-vaccine-from-AstraZeneca

[4] Public Health Agency of Canada. NACI rapid response: Interchangeability of authorized COVID-19 vaccines. Ottawa: Public Health Agency of Canada; 2021. Available from: https://www.canada.ca/en/public-health/services/immunization/national-advisory-committee-on-immunization-naci/recommendations-use-covid-19-vaccines/rapid-response-interchangeability.html

[5] Shaw RH Stuart A Greenland M Liu X Nguyen Van-Tam JS Snape MD Heterologous prime-boost COVID-19 vaccination: initial reactogenicity data. Lancet. 2021;397(10289):2043-6. 10.1016/S0140-6736(21)01115-6 33991480 PMC8115940

[6] NHS England. COVID-19 Vaccinations. London: NHS England; 2021. Available from: https://www.england.nhs.uk/statistics/statistical-work-areas/covid-19-vaccinations/

[7] R Core Team. R: A Language and Environment for Statistical Computing. R Foundation for Statistical Computing; 2020

[8] Our World in Data. Coronavirus (COVID-19) Vaccinations. Oxford: Our World in Data; 2021. Available from: https://ourworldindata.org/covid-vaccinations

[9] Public Health England. SARS-CoV-2 variants of concern and variants under investigation in England. London: Public Health England; 2021. Available from: https://assets.publishing.service.gov.uk/government/uploads/system/uploads/attachment_data/file/993879/Variants_of_Concern_VOC_Technical_Briefing_15.pdf

[10] Polack FP Thomas SJ Kitchin N Absalon J Gurtman A Lockhart S Safety and Efficacy of the BNT162b2 mRNA Covid-19 Vaccine. N Engl J Med. 2020;383(27):2603-15. 10.1056/NEJMoa2034577 33301246 PMC7745181

[11] Folegatti PM Ewer KJ Aley PK Angus B Becker S Belij-Rammerstorfer S Safety and immunogenicity of the ChAdOx1 nCoV-19 vaccine against SARS-CoV-2: a preliminary report of a phase 1/2, single-blind, randomised controlled trial. Lancet. 2020;396(10249):467-78. 10.1016/S0140-6736(20)31604-4 32702298 PMC7445431

[12] Schmidt T, Klemis V, Schub D, Mihm J, Hielscher F, Marx S, et al. Immunogenicity and reactogenicity of a heterologous COVID-19 prime-boost vaccination compared with homologous vaccine regimens. medRxiv. 2021: 2021.06.13.21258859.

[13] Groß R, Zanoni M, Seidel A, Conzelmann C, Gilg A, Krnavek D, et al. Heterologous ChAdOx1 nCoV-19 and BNT162b2 prime-boost vaccination elicits potent neutralizing antibody responses and T cell reactivity. medRxiv. 2021: 2021.05.30.21257971. 10.1016/j.ebiom.2021.103761 PMC8682749 34929493

[14] Hillus D, Schwarz T, Tober-Lau P, Hastor H, Thibeault C, Kasper S, et al. Safety, reactogenicity, and immunogenicity of homologous and heterologous prime-boost immunisation with ChAdOx1-nCoV19 and BNT162b2: a prospective cohort study. medRxiv. 2021: 2021.05.19.21257334. 10.1016/S2213-2600(21)00357-X PMC8360702 34391547

[15] Borobia AM Carcas AJ Pérez-Olmeda M Castaño L Bertran MJ García-Pérez J Immunogenicity and reactogenicity of BNT162b2 booster in ChAdOx1-S-primed participants (CombiVacS): a multicentre, open-label, randomised, controlled, phase 2 trial. Lancet. 2021;398(10295):121-30. 10.1016/S0140-6736(21)01420-3 34181880 PMC8233007

[16] Spencer AJ McKay PF Belij-Rammerstorfer S Ulaszewska M Bissett CD Hu K Heterologous vaccination regimens with self-amplifying RNA and adenoviral COVID vaccines induce robust immune responses in mice. Nat Commun. 2021;12(1):2893. 10.1038/s41467-021-23173-1 34001897 PMC8129084

