# Erratum for Euro Surveill. 2021;26(28)

**DOI:** 10.2807/1560-7917.ES.2022.27.8.220224e

**Published:** 2022-02-24

**Authors:** Eurosurveillance editorial team

**Affiliations:** 1 European Centre for Disease Prevention and Control (ECDC), Stockholm, Sweden

In the article ‘ Real-world data shows increased reactogenicity in adults after heterologous compared to homologous prime-boost COVID-19 vaccination, March−June 2021, England’ by Powell et al. published on 15 July 2021, six instances of Vaxzevria were misspelled in the original publication. These errors were corrected on 24 February 2022. We apologise for any inconvenience these typos may have caused.

