# Supplementary material for: Real-world data shows increased reactogenicity in adults after heterologous compared to homologous prime-boost COVID-19 vaccination, March−June 2021, England
Source: Euro Surveill. 2021 Jul 15;26(28):2100634. doi: 10.2807/1560-7917.ES.2021.26.28.2100634 (PMC8284043; doi:10.2807/1560-7917.ES.2021.26.28.2100634)
Supplement: Supplementary Material [file 21-00634_AMIRTHALINGAM_Supplement.pdf]

This supplementary material is hosted by *Eurosurveillance* as supporting information alongside the article Real-world data shows increased reactogenicity in adults after heterologous compared to homologous prime-boost COVID-19 vaccination, March-June 2021, England, on behalf of the authors who remain responsible for the accuracy and appropriateness of the content. The same standards for ethics, copyright, attributions and permissions as for the article apply. Supplements are not edited by Eurosurveillance and the journal is not responsible for the maintenance of any links or email addresses provided therein.

S1. Participant Questionnaire

Questionnaire One

Your first name(s)	Your last name	Date of Birth
**Address**		
		Postcode:
**Telephone number**	**Email address**	

Today's date: D D / M M / Y Y

Gender: ☐Male ☐ Female ☐Prefer not to answer

If you are currently working, please state your occupation

☐ Frontline HCW (Hospital) ☐ Frontline HCW (Community) ☐ Social Care Worker

☐ Care home worker ☐ Other (please specify) ____________________

☐ Not currently working

Which one of the following groups do you belong to?

**White**	**Asian / Asian British**
☐ English / Welsh / Scottish / Northern Irish / British	☐ Indian
☐ Irish	☐ Pakistani
☐ Gypsy or Irish Traveller	☐ Bangladeshi
☐ Any other White background	☐ Chinese
	☐ Any other Asian background
**Mixed / Multiple ethnic groups**	
☐ White and Black Caribbean	
☐ White and Black African	

☐ White and Asian<br>☐ Any other Mixed / Multiple ethnic background<br><br>**Black / African / Caribbean / Black British**<br>☐ African<br>☐ Caribbean<br>☐ Any other Black / African / Caribbean	**Other ethnic group**<br>☐ Arab<br>☐Any other ethnic group *(state below):*<br><br><br><br>☐ Prefer not to answer

Have you got any medical conditions? ☐ yes ☐ no ☐ not known

If **yes**, please give details:

Did you have two different COVID-19 vaccines? ☐ yes ☐ no ☐ not known

Vaccine and dose	Date of vaccination	Name of vaccine	Batch number
COVID-19 dose 1	D D / M M / Y Y		
COVID-19 dose 2	D D / M M / Y Y		

Why did you have a different vaccine brand for your second dose?

☐ I had a bad reaction to my first vaccine
☐ It was the only vaccine they had for my second dose
☐ I wanted to make sure I got this vaccine for my second dose
☐ I don’t know why I got a different vaccine
☐ It was given to me by mistake
☐ Other reason – please describe below:

Household Composition

**a.**	Number of children under 18 years in your household:	
**b.**	Number of adults aged 18-69 years in your household:	
**c.**	Number of older adults aged 70 years & over in your household:	

HISTORY OF COVID-19 RELATED ILLNESS OR SYMPTOMS

Have you had any COVID-19 related symptoms in the past year?

☐ Yes, I had symptoms but I was not tested
☐ Yes, I had symptoms but my test(s) were all negative
☐ Yes, I had symptoms and I had at least one positive test
☐ Yes, I had symptoms, had a test, but it failed
☐ No
☐ I am not sure

If yes, when did your COVID-19 related symptoms start? D D / M M / Y Y

Approximate dates are fine. If you have had symptoms more than once, record the first date.

If yes, how long were you ill for? ________________ days

If yes, were you admitted to hospital? ☐ yes ☐ no ☐ not known

If you had a positive COVID-19 result, when was that positive sample taken?

D D / M M / Y Y

Has anyone else in your household tested positive for COVID-19?

☐ no ☐ yes If yes, approximate number of people ☐

In the last 14 days have you had any contact with a person with confirmed COVID-19?

☐ no ☐ yes If yes, date of exposure D D / M M / Y Y

Post Vaccination Symptoms

Did you develop any symptoms after dose 1? ☐ yes ☐ no ☐ not known

IF yes, for each of the symptoms listed below, please use the scale below to grade any symptoms for this dose

☐ 0 No symptoms
☐ 1 Mild – easily tolerated with no limitation on normal activity
☐ 2 Moderate – some limitation of daily activity
☐ 3 Severe – unable to perform normal daily activity
☐ 4 Emergency department or hospital admission required

DOSE 1 SYMPTOMS	Date symptom started	Scale (0-4) on worst day	How long did your symptoms last? (in days)
Fever			
Chills			
Headache			
Generally unwell			
Tiredness			
Joint pain /aches			
Nausea /Vomiting			
**REACTION AT INJECTION SITE**			
Pain			

Tenderness			
Itching			
Redness / Warmth			
Other			

Did you develop any symptoms after dose 2? ☐ yes ☐ no ☐ not known

IF yes, for each of the symptoms listed below, please use the scale below to grade any symptoms for this dose

☐ 0 No symptoms
☐ 1 Mild – easily tolerated with no limitation on normal activity
☐ 2 Moderate – some limitation of daily activity
☐ 3 Severe – unable to perform normal daily activity
☐ 4 Emergency department or hospital admission required

**DOSE 2 SYMPTOMS**	Date symptom started	Scale (0-4) on worst day	How long did your symptoms last? (in days)
Fever			
Chills			
Headache			
Generally unwell			
Tiredness			
Joint pain /aches			
Nausea /Vomiting			
**REACTION AT INJECTION SITE**			
Pain			
Tenderness			
Itching			
Redness / Warmth			
Other			

Thank you for taking the time to complete the questionnaire.

S2. Demographics by vaccination schedule

Table showing the demographics of participants included in the analysis by vaccination schedule

	ChAd/ChAd		ChAd/BNT		BNT/ChAd		BNT/BNT	
Sex	n	%	n	%	n	%	n	%
Male	179	38.8	131	22.9	58	34.7	32	28.3
Female	282	61.2	437	76.4	108	64.7	81	71.7
Non-binary	-	-	1	0.2	-	-	-	-
Not known	-	-	2	0.3	-	-	-	-
Age category	Median age (IQR)		Median age (IQR)		Median age (IQR)		Median age (IQR)	
Under 50	40 (32-45)		38 (32-45)		41 (35-46)		33 (29-42)	
50 and over	54 (52-58)		59 (54-65)		63 (58-67)		59 (55-64)	
Ethnicity	n	%	n	%	n	%	n	%
White	399	86.6	515	90	148	88.6	88	77.9
Black	21	4.6	7	1.2	3	1.8	5	4.4
Asian	28	6.1	24	4.2	10	6	15	13.3
Mixed	9	2	17	3	3	2	3	2.7
Other	1	0.2	6	1	3	1.8	1	0.9
Unknown	3	0.7	3	0.5	-	-	1	0.9
Previous infection								
Confirmed infection	73	15.8	29	5	13	7.8	12	10.6
Previous symptoms	101	21.9	153	26.7	29	17.4	17	15
No prior infection	287	3	390	68.2	125	74.9	84	74.3
Total	461	35.1	572	43.6	167	12.7	113	8.6

BNT: Comirnaty (BNT162b2, BioNTech-Pfizer, Mainz, Germany/New York, United States (US); ChAd: Vaxrevia (ChAdOx1/nCoV-19, AstraZeneca, Cambridge, United Kingdom (UK).

S.3 Vaccination schedule by previous infection

Number of participants who reported local and systemic symptoms after prime and boost vaccines by vaccination schedule and previous infection

a) Those who reported no previous infection or symptoms of COVID-19

	Prime								Boost							
	ChAd/ChAd (n=287)		ChAd/BNT (n=390)		BNT/ChAd (n=125)		BNT/BNT (n=84)		ChAd/ChAd (n=287)		ChAd/BNT (n=390)		BNT/ChAd (n=125)		BNT/BNT (n=84)	
	n	% (95%CI)	n	% (95%CI)	n	% (95%CI)	n	% (95%CI)	n	% (95%CI)	n	% (95%CI)	n	% (95%CI)	n	% (95%CI)
Systemic																
Fever	104	36.2(30.6-42.0)	195	50.0(45.1-55.2)	16	12.8(7.5-20.0)	6	7.1(2.7-14.9)	17	5.9(3.5-9.3)	52	13.3(10.1-17.2)	34	27.2(19.6-35.9)	6	7.1(2.7-14.9)
Chills	119	41.5(35.7-47.4)	197	50.5(45.6-55.7)	13	10.4(5.70-17.1)	6	7.1(2.7-14.9)	24	8.4(5.4-12.2)	72	18.5(17.8-22.7)	36	28.8(21.1-37.6)	7	8.3(3.4-16.4)
Headache	109	38.0(32.3-43.9)	237	60.8(55.9-65.8)	22	17.6(11.4-25.4)	12	14.3(7.6-23.6)	48	16.7(12.6-21.6)	119	30.5(26.0-35.4)	44	35.2(26.9-44.2)	12	14.3(7.6-23.6)
Unwell	141	49.1(43.2-55.1)	259	66.4(61.7-71.3)	29	23.2(16.1-31.6)	13	15.5(8.5-25.0)	53	18.5(14.1-23.4)	119	30.5(26.0-35.4)	48	38.4(29.8-47.5)	15	17.9(10.4-27.7)
Tiredness	138	48.1(42.2-54.0)	254	65.1(60.3-70.0)	29	23.2(16.1-31.6)	15	17.9(10.4-27.7)	60	20.9(16.4-26.1)	129	33.1(28.5-38.1)	47	37.6(29.1-46.7)	19	22.6(14.2-33.0)
Joint pain	105	36.6(31.0-42.5)	190	48.7(43.8-53.9)	20	16.0(10.6-23.6)	7	8.3(3.4-16.4)	37	12.9(9.2-17.3)	74	19.0(15.2-23.3)	36	28.8(21.1-37.6)	9	10.7(5.0-19.4)
Nausea	27	9.4(6.3-13.4)	99	25.4(21.2-30.1)	12	9.6(5.1-16.2)	1	1.2(0.0-6.5)	5	1.7(0.6-4.0)	34	8.7(6.1-12.0)	10	8.0(3.9-14.2)	1	1.2(0.0-6.5)
Local																
Pain	117	40.8(35.0-46.7)	199	51.0(46.1-56.2)	30	24.0(16.8-32.5)	21	25.0(16.2-35.6)	57	19.9(15.4-25.0)	147	37.7(33.0-42.8)	40	32.0(23.9-40.9)	17	20.2(12.3-30.4)
Tenderness	113	39.4(33.7-45.3)	184	47.1(42.2-52.4)	27	21.6(14.7-29.9)	23	27.4(18.2-38.2)	57	19.9(15.4-25.0)	149	38.2(33.4-43.3)	42	33.6(25.4-42.6)	20	23.8(15.2-34.3)
Itch	13	4.5(2.4-7.6)	25	6.4(4.2-9.3)	7	5.6(2.3-11.2)	1	1.2(0.0-6.5)	9	3.1(1.4-5.90)	15	3.9(2.2-6.2)	5	4.0(1.3-9.1)	1	1.2(0.0-6.5)
Redness	35	12.2(8.6-16.6)	71	18.2(14.5-22.5)	11	8.8(4.5-15.2)	4	4.8(1.3-11.7)	10	3.5(1.7-6.3)	38	9.7(7.0-13.2)	14	11.2(6.3-18.1)	1	1.2(0.0-6.5)
Total																
Systemic	180	62.7(56.8-68.3)	300	76.9(72.6-81.2)	41	32.8(24.7-41.8)	21	25.0(16.2-35.6)	82	28.6(23.4-34.2)	177	45.4(40.5-50.6)	62	49.6(40.5-58.7)	24	28.6(19.2-39.5)
Local	142	49.5(43.6-55.4)	277	58.2(66.4-75.7)	38	30.4(22.5-39.3)	25	29.8(20.3-40.7)	73	25.4(20.5-30.9)	179	45.9(41.0-51.1)	49	39.2(30.6-48.3)	25	29.8(20.3-40.7)
Overall	183	63.8(57.9-69.3)	313	80.5(76.2-84.3)	53	42.4(33.6-51.6)	28	33.3(23.4-44.5)	96	33.5(28.0-39.2)	212	54.4(49.4-59.5)	69	55.2(46.1-64.1)	28	33.3(23.4-44.5)
Medical attention	19	6.6(4.0-10.2)	127	32.6(28.0-37.6)	22	17.6(11.4-25.4)	4	4.8(1.3-11.8)	8	2.8(1.2-5.4)	28	7.2(4.8-10.2)	19	14.4(8.8-21.8)	5	6.0(2.0-13.4)

b) Those who reported confirmed SARS-CoV-2 infection

	Prime								Boost							
	ChAd/ChAd (n=73)		ChAd/BNT (n=29)		BNT/ChAd (n=13)		BNT/BNT (n=12)		ChAd/ChAd (n=73)		ChAd/BNT (n=29)		BNT/ChAd (n=13)		BNT/BNT (n=12)	
	n	% (95%CI)	n	% (95%CI)	n	% (95%CI)	n	% (95%CI)	n	% (95%CI)	n	% (95%CI)	n	% (95%CI)	n	% (95%CI)
Systemic																
Fever	34	46.6(34.8-58.6)	17	58.6(38.9-76.5)	5	38.5(13.9-68.4)	4	33.3(9.9-65.1)	3	4.1(0.7-11.5)	8	27.6(12.7-47.2)	1	7.7(0.2-36.0)	3	25.0(5.5-57.2)
Chills	34	46.6(34.8-58.6)	9	31.0(15.3-50.8)	4	30.8(9.1-61.4)	4	33.3(9.9-65.1)	3	4.1(0.7-11.5)	6	20.7(8.0-39.7)	2	15.4(1.9-45.4)	2	16.7(2.1-48.4)
Headache	37	50.7(38.7-62.6)	17	58.6(38.9-76.5)	7	53.9(25.1-80.8)	7	58.3(27.7-84.8)	16	21.9(13.1-33.1)	13	44.8(26.4-64.3)	8	61.5(31.6-86.1)	3	25.0(5.5-57.2)
Unwell	43	58.9(46.8-70.3)	20	69.0(49.2-84.7)	7	53.9(25.1-80.8)	8	66.7(34.9-90.1)	10	13.7(6.7-23.8)	13	44.8(26.4-64.3)	5	38.5(13.86-68.42)	4	33.3(9.9-65.1)
Tiredness	43	58.9(46.8-70.3)	21	72.4(52.8-87.8)	7	53.9(25.1-80.8)	7	58.3(27.7-84.8)	14	19.2(10.9-30.1)	14	48.3(29.4-67.5)	7	53.9(25.1-80.8)	5	41.7(15.2-72.3)
Joint pain	32	43.8(32.2-56.0)	17	58.6(38.9-76.5)	4	30.8(9.1-61.4)	4	33.3(9.9-65.1)	6	8.2(3.1-17.0)	9	31.0(15.3-50.8)	5	38.5(13.9-68.4)	3	25.0(5.5-57.2)
Nausea	8	11.0(4.9-20.5)	4	13.8(3.9-31.7)	2	15.4(1.9-45.4)	1	8.3(0.2-38.5)	1	1.4(0.0-7.4)	5	17.2(5.8-35.8)	2	15.4(1.9-45.4)	1	8.3(00.2-38.5)
Local																
Pain	38	52.1(40.0-64.0)	17	58.6(38.9-76.5)	7	53.9(25.1-80.8)	8	66.7(34.9-90.1)	8	11.0(4.9-20.5)	10	34.5(17.9-54.3)	7	53.9(25.1-80.8)	4	33.3(9.9-65.1)
Tenderness	37	50.7(38.7-62.6)	14	48.3(29.4-67.5)	5	38.5(13.9-68.4)	7	58.3(27.7-84.8)	7	9.6(3.9-18.8)	14	48.3(29.4-67.5)	5	38.5(13.9-68.4)	5	41.7(15.2-72.3)
Itch	4	5.5(1.5-13.4)	3	10.3(21.9-27.4)	3	23.1(5.0-53.8)	1	8.3(00.2-38.5)	1	1.4(0.0-7.4)	3	10.3(21.9-27.4)	0	0(0-24.7)	2	16.7(2.1-48.4)
Redness	16	21.9(13.1-33.1)	8	27.6(12.7-47.2)	3	23.1(5.0-53.8)	4	33.3(9.9-65.1)	7	9.6(3.9-18.8)	8	27.6(12.7-47.3)	3	23.1(5.0-53.8)	3	25.0(5.5-57.2)
Total																
Systemic	53	72.6(60.9-82.4)	23	79.3(60.3-92.0)	9	69.2(38.6-90.9)	8	66.7(34.9-90.1)	19	26.0(16.5-37.6)	17	58.6(38.9-76.5)	10	76.9(46.2-95.0)	5	41.7(15.2-72.3)
Local	47	64.4(52.3-75.3)	17	58.6(38.9-76.5)	9	69.2(38.6-90.9)	8	66.7(34.9-90.1)	12	16.4(8.8-27.0)	15	51.7(32.5-70.6)	7	53.9(25.1-80.8)	5	41.7(15.2-72.3)
Overall	53	72.6(60.9-82.4)	23	79.3(60.3-92.0)	9	69.2(38.6-90.9)	9	75.0(42.8-94.5)	19	26.0(16.5-37.6)	18	62.1(42.3-79.3)	10	76.9(46.2-9.5)	5	41.7(15.2-72.3)
Medical attention	10	13.7(6.8-23.8)	9	31.0(15.3-50.8)	6	46.2(19.2-74.9)	1	8.3(0.2-38.5)	2	2.7(0.3-9.6)	3	10.3(2.2-27.4)	3	23.1(5.0-53.8)	1	8.3(0.2-38.5)

c) Those who reported symptoms of COVID-19 but no confirmatory test

	Prime								Boost							
	ChAd/ChAd (n=101)		ChAd/BNT (n=153)		BNT/ChAd (n=29)		BNT/BNT (n=17)		ChAd/ChAd		ChAd/BNT		BNT/ChAd		BNT/BNT	
	n	% (95%CI)	n	% (95%CI)	n	% (95%CI)	n	% (95%CI)	n	% (95%CI)	n	% (95%CI)	n	% (95%CI)	n	% (95%CI)
Systemic																
Fever	50	49.5(39.4-59.6)	104	68.0(60.0-75.3)	9	31.0(15.3-50.8)	3	17.7(3.8-43.4)	9	8.9(4.2-16.3)	33	21.6(15.3-28.9)	11	37.9(20.68-57.7)	3	17.7(3.8-43.4)
Chills	43	42.6(32.8-52.8)	90	58.8(50.6-66.7)	4	13.8(3.9-31.7)	5	29.4(10.3-56.0)	13	12.9(7.0-21.0)	30	19.6(13.6-26.8)	8	27.6(12.7-47.2)	3	17.7(3.8-43.4)
Headache	54	53.5(43.3-63.5)	116	75.8(68.2-82.4)	8	27.6(12.7-47.2)	5	29.4(10.3-56.0)	29	28.7(20.1-38.6)	63	41.2(33.3-49.4)	18	62.1(42.3-79.3)	3	17.7(3.8-43.4)
Unwell	60	59.4(49.2-69.1)	126	82.4(75.4-88.0)	13	44.8(26.4-64.3)	7	41.2(18.4-67.1)	21	20.8(13.4-30.0)	67	43.8(35.8-52.0)	17	58.6(38.9-76.5)	3	17.7(3.8-43.4)
Tiredness	60	59.4(49.2-69.1)	128	83.7(76.8-89.1)	11	37.9(20.7-57.7)	6	35.3(14.2-61.7)	32	31.7(22.8-41.7)	78	51.0(42.8-59.1)	18	62.1(42.3-79.3)	2	11.8(1.5-36.4)
Joint pain	47	46.5(36.6-56.7)	91	59.5(51.3-67.3)	3	10.3(2.2-27.4)	6	35.3(14.2-61.7)	16	15.8(9.3-24.5)	47	30.7(23.5-38.7)	9	31.0(15.3-50.8)	1	5.9(0.2-28.7)
Nausea	14	13.9(7.8-22.2)	50	32.7(25.3-40.7)	6	20.7(8.0-39.7)	2	11.8(1.5-36.4)	8	7.9(3.5-15.0)	19	12.4(7.6-18.7)	9	31.0(15.3-50.8)	1	5.9(0.2-28.7)
Local																
Pain	48	47.5(37.5-57.7)	90	58.8(50.6-66.7)	12	41.4(23.5-61.1)	6	35.3(14.2-61.7)	23	22.8(15.0-32.2)	78	51.0(42.8-59.1)	17	58.6(38.9-76.5)	3	17.7(3.8-43.4)
Tenderness	47	46.5(36.6-56.7)	94	61.4(53.2-69.2)	12	41.4(23.5-61.1)	7	41.2(18.4-67.1)	25	24.8(16.7-34.3)	90	58.8(50.6-66.7)	18	62.1(42.3-79.3)	3	17.7(3.8-43.4)
Itch	10	9.9(4.9-17.5)	13	8.5(4.6-14.1)	3	10.3(2.2-27.4)	1	5.9(0.2-28.7)	4	4.0(1.1-9.8)	7	4.6(1.9-9.2)	2	6.9(0.9-22.8)	0	0(0-19.5)
Redness	16	16.0(9.3-24.5)	38	24.8(18.2-32.5)	5	17.2(5.8-35.8)	2	11.8(1.5-36.4)	6	5.9(2.2-12.5)	25	16.3(10.9-23.8)	4	13.8(3.9-31.7)	1	5.9(0.2-28.7)
Total																
Systemic	70	69.3(59.3-78.1)	137	89.5(83.6-93.9)	16	55.2(35.7-73.6)	9	52.9(27.8-77.0)	46	45.5(35.6-55.8)	98	64.1(55.9-71.6)	19	65.5(45.7-82.1)	6	35.3(14.2-61.7)
Local	57	56.4(46.2-66.3)	105	68.6(60.6-75.9)	14	48.3(29.5-67.5)	9	52.9(27.8-77.0)	34	33.7(24.6-43.8)	103	67.3(59.3-74.7)	18	62.1(42.3-79.3)	4	23.5(6.8-49.9)
Overall	71	70.3(60.4-79.0)	138	90.8(84.3-94.4)	18	62.1(42.3-79.3)	9	52.9(27.8-77.0)	47	47.0(36.6-56.7)	116	75.8(68.7-82.8)	19	65.5(45.7-82.1)	6	35.3(14.2-61.7)
Medical attention	5	5.0(1.6-11.2)	52	34.0(26.5-42.1)	7	24.1(10.2-43.5)	3	17.7(3.8-43.4)	3	3.0(6.2-8.4)	24	15.7 (10.3-24.4)	10	34.5(17.9-54.3)	1	5.9(0.2-28.7)

BNT: Comirnaty (BNT162b2, BioNTech-Pfizer, Mainz, Germany/New York, United States (US); ChAd: Vaxrevia (ChAdOx1/nCoV-19, AstraZeneca, Cambridge, United Kingdom (UK).

Please note individuals could report more than one reaction therefore totals do not match the sum of the symptoms.

S.4 Vaccination schedule by age group

Number of participants who reported local and systemic symptoms after prime and boost vaccines by vaccination group and age and who did not report previous symptoms of COVID-19 nor confirmed infection

a) Those aged under 50

	Prime								Boost							
	ChAd/ChAd (n=85)		ChAd/BNT (n=189)		BNT/ChAd (n=38)		BNT/BNT (n=37)		ChAd/ChAd (n=85)		ChAd/BNT (n=189)		BNT/ChAd (n=38)		BNT/BNT (n=37)	
	n	% (95%CI)	n	% (95%CI)	n	% (95%CI)	n	% (95%CI)	n	% (95%CI)	n	% (95%CI)	n	% (95%CI)	n	% (95%CI)
Systemic																
Fever	43	50.6(39.5-61.6)	109	57.7(50.3-64.9)	5	13.1(4.4-28.1)	5	13.5(4.5-28.8)	10	11.8(57.9-20.6)	26	13.8(9.2-19.5)	17	44.7(28.2-61.7)	5	13.5(4.5-28.8)
Chills	46	54.1(43.0-65.0)	110	58.2(50.8-65.3)	5	13.1(4.4-28.1)	4	10.8(3.0-25.4)	10	11.8(57.9-20.6)	33	17.5(12.3-23.6)	17	44.7(28.2-61.7)	4	10.8(3.0-25.4)
Headache	42	49.4(38.4-60.5)	120	63.5(56.2-70.4)	7	18.4(7.7-34.3)	7	18.9(8.0-35.2)	19	22.4(14.0-32.7)	65	34.4(27.7-41.6)	17	44.7(28.2-61.7)	9	24.3(11.8-41.2)
Unwell	49	57.7(46.4-68.3)	138	73.0(66.1-79.2)	7	18.4(7.7-34.3)	6	16.2(61.9-32.0)	19	22.4(14.0-32.7)	65	34.4(27.7-41.6)	23	60.5(43.4-76.0)	9	24.3(11.8-41.2)
Tiredness	49	57.7(46.4-68.3)	134	70.9(63.9-77.3)	9	23.7(11.4-40.2)	11	29.7(15.9-470)	20	23.5(15.0-34.0)	73	38.6(31.7-46.0)	20	52.6(35.8-69.0)	10	27.0(13.8-44.1)
Joint pain	36	42.4(31.7-53.6)	103	54.5(47.1-61.7)	5	13.1(4.4-28.1)	4	10.8(3.0-25.4)	13	15.3(8.4-24.7)	41	21.7(16.0-28.3)	15	39.5(24.0-56.6)	5	13.5(4.5-28.8)
Nausea	12	14.1(7.5-23.4)	55	29.1(22.7-36.1)	5	13.1(4.4-28.1)	0	0(0-9.5)	1	1.2(0.0-6.4)	14	7.4(4.1-12.1)	6	16.0(6.0-31.3)	1	2.7(0.1-14.2)
Local																
Pain	44	51.8(40.7-62.7)	108	57.1(49.8-64.3)	8	21.1(9.6-37.3)	13	35.1(20.2-52.5)	21	24.7(16.0-35.3)	74	39.2(32.2-46.5)	19	50.0(33.4-66.6)	7	18.9(8.0-35.2)
Tenderness	41	48.2(37.3-59.3)	94	49.7(42.4-57.1)	8	21.1(9.6-37.3)	13	35.1(20.2-52.5)	19	22.4(14.0-32.7)	74	39.2(32.2-46.5)	17	44.7(28.2-61.7)	10	27.0(13.8-44.1)
Itch	7	8.2(3.4-16.2)	14	7.4(4.1-12.1)	3	7.9(16.6-21.4)	0	0(0-9.5)	1	1.2(0.0-6.4)	9	4.8(2.2-8.8)	3	7.9(16.6-21.4)	0	0(0-9.5)
Redness	19	22.4(14.0-32.7)	38	20.1(14.6-29.5)	5	13.1(4.4-28.1)	1	2.7(0.06-14.16)	3	1.2(0.0-6.4)	13	6.9(3.7-11.5)	5	13.2(4.4-28.1)	0	0(0-9.5)
Total																
Systemic	63	74.1(63.5-83.0)	153	82.0(74.6-86.3)	10	26.3(13.4-43.1)	13	35.1(20.2-52.5)	28	32.9(23.1-44.0)	92	48.7(41.4-56.0)	25	65.8(48.7-80.4)	13	35.1(20.2-52.5)
Local	52	61.2(50.0-71.6)	120	63.5(56.2-70.4)	9	23.8(11.4-40.2)	15	40.5(24.7-57.9)	26	30.6(21.0-41.5)	91	48.2(40.8-55.5)	21	55.3(38.3-71.4)	12	32.4(18.0-49.8)
Overall	63	74.1(63.5-83.0)	156	82.5(76.4-87.7)	14	36.8(21.8-54.0)	16	43.2(27.1-60.5)	32	37.7(27.4-48.8)	108	57.1(49.8-64.3)	29	76.3(59.8-88.6)	14	37.8(22.5-55.2)
Medical attention	7	8.2(3.4-16.2)	60	31.8(25.2-38.9)	9	23.8(11.4-40.2)	2	5.4(0.7-18.2)	4	4.7(1.3-11.6)	11	5.8(2.9-10.2)	10	26.3(13.4-43.1)	1	2.7(0.1-14.2)

b) Those aged 50 and over

	Prime								Boost							
	ChAd/ChAd (n=202)		ChAd/BNT (n=201)		BNT/ChAd (n=87)		BNT/BNT (n=47)		ChAd/ChAd (n=202)		ChAd/BNT (n=201)		BNT/ChAd (n=87)		BNT/BNT (n=47)	
	n	% (95%CI)	n	% (95%CI)	n	% (95%CI)	n	% (95%CI)	n	% (95%CI)	n	% (95%CI)	n	% (95%CI)	n	% (95%CI)
Systemic																
Fever	61	30.2(24.0-37.0)	86	42.8(35.8-49.9)	11	12.6(6.5-21.5)	1	2.1(0.1-11.3)	7	3.5(1.4-7.0)	26	12.9(8.6-18.4)	17	19.5(11.8-29.4)	1	2.1(0.1-11.3)
Chills	73	36.0(29.5-43.2)	87	43.3(36.3-50.4)	8	9.2(4.1-17.3)	2	4.3(0.5-14.5)	14	6.9(3.8-11.4)	39	19.4(14.2-25.6)	19	21.8(13.7-32.0)	3	6.4(1.3-17.5)
Headache	67	33.2(26.7-40.1)	117	58.2(51.1-65.1)	15	17.2(10.0-26.8)	5	10.6(3.5-23.1)	29	14.4(9.8-20.0)	54	26.9(20.9-33.6)	27	31.0(21.5-41.9)	3	6.4(1.3-17.5)
Unwell	92	45.5(38.5-52.7)	121	60.2(53.1-67.0)	22	25.3(16.6-35.7)	7	14.9(6.2-28.3)	34	18.8(12.0-22.7)	54	26.9(20.9-33.6)	25	28.7(19.5-39.4)	6	12.8(4.8-25.7)
Tiredness	89	44.1(37.1-51.2)	120	59.7(52.6-66.5)	20	23.0(14.6-33.2)	4	8.5(2.4-20.4)	40	19.8(14.5-26.0)	56	27.9(21.8-34.6)	27	31.0(21.5-41.9)	9	19.6(9.1-33.3)
Joint pain	69	34.2(27.7-41.1)	87	43.28(36.3-50.4)	15	17.2(10.0-26.8)	3	6.4(1.3-17.5)	24	11.9(7.8-17.2)	33	16.4(11.6-22.3)	21	24.1(15.6-34.5)	4	8.5(2.4-20.4)
Nausea	15	7.4(4.2-12.0)	44	21.9(16.3-28.3)	7	8.1(3.3-15.9)	1	2.1(0.1-11.3)	4	2.0(0.5-5.0)	20	10.0(6.2-15.0)	4	4.6(1.3-11.4)	0	0(0-0.8)
Local																
Pain	73	36.1(29.5-43.2)	91	45.3(38.3-52.30	22	25.3(16.6-35.7)	8	17.0(7.7-30.8)	36	17.8(12.8-23.8)	73	36.3(29.7-43.4)	21	24.1(15.6-34.5)	10	21.3(10.7-35.7)
Tenderness	72	35.6(29.0-42.7)	90	44.9(37.8-51.9)	19	21.8(13.7-32.0)	10	21.3(10.7-35.7)	38	18.8(13.6-24.9)	75	37.3(30.6-44.4)	25	28.7(19.5-39.4)	10	21.3(10.7-35.7)
Itch	6	3.0(1.1-6.4)	11	5.5(2.8-9.6)	4	4.6(1.3-11.4)	1	2.1(0.1-11.3)	8	4.0(1.7-7.7)	6	3.0(1.1-6.4)	2	2.3(0.3-8.6)	1	2.1(0.1-11.3)
Redness	16	7.9(4.6-64.8)	33	16.4(11.6-22.3)	6	6.9(2.6-14.4)	3	6.4(1.3-17.5)	7	3.5(1.4-7.0)	25	12.4(8.2-17.8)	9	10.3(4.8-18.7)	1	2.1(0.1-11.3)
Total																
Systemic	117	57.9(50.8-64.8)	147	73.1(66.4-79.1)	31	35.6(35.6-46.6)	8	17.0(7.7-30.8)	54	26.7(20.8-33.4)	85	42.3(35.4-49.4)	37	42.5(32.0-53.6)	11	23.4(12.3-38.0)
Local	90	44.6(37.6-51.6)	107	53.2(46.1-60.3)	29	33.3(23.6-44.3)	10	21.3(10.7-35.7)	47	23.3(17.6-29.7)	88	43.8(36.8-50.9)	28	32.2(22.6-43.1)	13	27.7(15.6-42.6)
Overalll	120	59.4(43.4-57.6)	157	78.5(71.8-83.6)	39	44.8(31.6-55.9)	12	25.1(13.9-40.4)	64	31.7(25.3-38.6)	104	51.7(44.6-58.8)	40	46.0(35.2-57.0)	14	29.8(17.3-44.9)
Medical attention	12	5.9(3.1-10.6)	67	33.3(26.9-40.3)	13	14.9(8.2-24.2)	2	4.3(0.5-14.5)	4	2.0(0.5-5.0)	17	8.5(5.0-13.2)	9	9.2(4.1-17.3)	4	8.5(2.4-20.4)

BNT: Comirnaty (BNT162b2, BioNTech-Pfizer, Mainz, Germany/New York, United States (US); ChAd: Vaxrevia (ChAdOx1/nCoV-19, AstraZeneca, Cambridge, United Kingdom (UK).

Please note individuals could report more than one reaction therefore totals do not match the sum of the symptoms.

S.5 Vaccination schedule by sex

Number of participants who reported local and systemic symptoms after prime and boost vaccines by vaccination group and sex and who did not report previous symptoms of COVID-19 nor confirmed infection

a) Women

	Prime								Boost							
	ChAd/ChAd (n=176)		ChAd/BNT (n=296)		BNT/ChAd (n=76)		BNT/BNT (n=58)		ChAd/ChAd (n=176)		ChAd/BNT (n=296)		BNT/ChAd (n=76)		BNT/BNT (n=58)	
	n	% (95%CI)	n	% (95%CI)	n	% (95%CI)	n	% (95%CI)	n	% (95%CI)	n	% (95%CI)	n	% (95%CI)	n	% (95%CI)
Systemic																
Fever	67	38.1(30.9-45.7)	161	54.4(48.5-60.2)	15	19.7(11.5-30.5)	6	10.3(3.9-21.2)	14	8.0(4.4-13.0)	42	14.2(10.4-18.7)	24	31.6(21.4-43.3)	4	6.9(1.9-16.3)
Chills	79	44.9(37.4-52.6)	168	56.8(50.9-62.5)	13	17.1(9.4-27.5)	3	5.2(1.1-14.4)	19	10.8(6.6-16.3)	63	21.3(16.8-26.4)	26	34.2(23.7-46.0)	4	6.9(1.9-16.3)
Headache	77	43.8(36.3-51.4)	201	67.9(62.3-73.2)	20	26.3(16.9-37.7)	12	20.7(11.2-33.4)	39	22.2(16.3-29.0)	98	33.1(27.8-38.8)	34	44.7(33.3-56.6)	12	20.7(11.2-33.4)
Unwell	93	52.8(45.2-60.4)	214	72.3(6.8-73.3)	24	31.6(21.4-43.3)	10	17.2(8.6-29.4)	41	23.3(17.3-30.2)	97	32.8(27.5-38.4)	35	46.1(34.6-58.9)	11	19.0(10.0-31.4)
Tiredness	97	55.1(47.4-62.6)	211	71.3(65.8-76.4)	24	31.6(21.4-43.3)	14	24.1(13.9-37.2)	48	27.3(20.8-34.5)	106	35.8(27.5-38.4)	36	47.4(35.8-59.2)	15	25.9(15.3-39.0)
Joint pain	78	44.3(36.9-52.0)	163	55.1(49.2-60.8)	18	23.7(14.7-34.8)	7	12.1(5.0-23.3)	31	17.6(12.3-24.1)	64	21.6(17.1-26.8)	27	35.5(24.9-47.3)	7	12.1(5.0-23.3)
Nausea	22	12.5(8.0-18.3)	85	28.7(23.6-34.2)	11	14.5(7.5-24.4)	1	1.7(0.0-9.2)	5	2.8(9.3-6.5)	31	10.5(7.2-14.5)	9	11.8(5.6-21.3)	1	1.7(0.0-9.2)
Local																
Pain	84	47.7(40.2-55.4)	167	56.4(50.6-62.1)	24	31.6(21.4-43.3)	18	31.0(19.5-44.5)	38	21.6(15.8-28.4)	120	40.5(34.9-46.4)	30	39.5(28.4-51.4)	12	20.7(11.2-33.4)
Tenderness	87	49.4(41.8-57.1)	159	53.7(47.9-59.5)	24	31.6(21.4-43.3)	20	34.5(22.5-48.1)	46	26.1(19.8-33.3)	131	44.3(38.5-50.1)	32	42.1(30.9-54.0)	17	29.3(18.1-42.7)
Itch	12	6.8(3.6-11.6)	24	8.1(5.3-11.8)	6	7.9(3.0-16.4)	1	1.7(0.0-9.2)	9	5.1(2.4-9.5)	12	4.1(2.1-7.0)	4	5.3(1.5-12.9)	1	1.7(0.0-9.2)
Redness	29	16.5(11.3-22.8)	64	21.6(17.1-27.8)	10	13.2(6.5-22.9)	4	6.9(1.9-16.3)	8	4.6(2.0-8.6)	34	11.5(8.1-15.7)	12	15.8(8.4-26.0)	1	1.7(0.0-9.2)
Total																
Systemic	118	67.1(60.0-73.9)	249	84.1(79.5-88.1)	33	43.4(32.1-55.3)	17	29.3(18.1-42.7)	64	36.4(29.3-43.9)	146	49.3(43.5-55.2)	44	57.9(46.0-69.1)	19	32.8(21.0-46.3)
Local	102	58.0(50.2-65.3)	89	63.9(58.1-69.3)	31	40.8(29.6-52.7)	21	36.2(24.0-50.0)	53	30.1(23.4-37.5)	149	50.3(44.5-56.2)	36	47.4(35.8-59.2)	19	32.8(21.0-46.3)
Overall	121	68.8(61.3-75.5)	252	85.4(80.6-89.0)	42	55.3(43.4-66.7)	23	39.7(27.0-53.4)	68	38.6(31.4-46.3)	176	59.5(56.6-65.1)	48	63.2(51.3-73.9)	21	36.2(24.0-50.0)
Medical attention	15	8.5(4.9-12.7)	103	34.8(29.4-40.5)	17	22.4(13.6-33.4)	3	5.2(1.1-14.4)	6	3.4(1.3-7.3)	25	8.5(5.5-12.2)	11	14.5(7.5-24.4)	3	5.2(1.1-14.4)

b) Men

	Prime								Boost							
	ChAd/ChAd (n=111)		ChAd/BNT (n=93)		BNT/ChAd (n=49)		BNT/BNT (n=26)		ChAd/ChAd (n=111)		ChAd/BNT (n=93)		BNT/ChAd (n=49)		BNT/BNT (n=26)	
	n	% (95%CI)	n	% (95%CI)	n	% (95%CI)	n	% (95%CI)	n	% (95%CI)	n	% (95%CI)	n	% (95%CI)	n	% (95%CI)
Systemic																
Fever	37	33.3(24.7-42.9)	34	36.5(26.8-47.2)	1	2.0(0.1-10.9)	0	0(0-13.2)	3	2.7(0.6-7.7)	10	10.8(5.3-18.9)	10	20.4(10.2-34.3)	2	7.7(0.9-25.1)
Chills	40	36.0(27.1-45.7)	28	30.1(21.0-40.5)	0	0.0(0-7.3)	3	11.5(2.4-30.2)	5	4.5(1.5-10.2)	9	9.7(4.5-17.6)	10	20.4(10.2-34.3)	3	11.5(2.4-30.2)
Headache	32	28.8(20.6-38.2)	35	37.6(27.8-48.3)	2	4.1(5.0-14.0)	0	0(0-13.2)	9	8.1(3.8-14.8)	20	21.5(13.7-31.2)	10	20.4(10.2-34.3)	0	0(0-13.2)
Unwell	48	43.2(33.9-53.0)	44	47.3(36.9-57.9)	5	10.2(3.4-22.2)	3	11.5(2.4-30.2)	12	10.8(5.7-18.1)	21	22.6(14.6-32.4)	13	26.5(15.0-41.1)	4	15.4(4.4-34.9)
Tiredness	41	36.9(28.0-46.6)	42	45.2(34.8-55.8)	5	10.2(3.4-22.2)	1	3.9(0.1-19.6)	12	10.8(5.7-18.1)	22	23.7(15.5-33.6)	11	22.5(11.8-36.6)	4	15.4(4.4-34.9)
Joint pain	27	24.3(16.7-33.4)	27	29.0(20.1-39.4)	2	4.1(5.0-14.0)	0	0(0-13.2)	6	5.4(2.0-11.4)	10	10.8(5.3-18.9)	9	18.4(8.8-32.0)	2	7.7(0.9-25.1)
Nausea	5	4.5(1.5-10.2)	13	14.0(7.7-22.7)	1	2.0(0.1-10.9)	0	0(0-13.2)	0	0(0-3.4)	3	3.2(0.7-9.1)	1	2.0(0.1-10.9)	0	0(0-13.23)
Local																
Pain	33	29.7(21.4-39.1)	31	33.3(23.9-43.9)	6	12.2(4.6-24.8)	3	11.5(2.4-30.2)	19	17.1(10.6-25.4)	27	29.0(20.1-39.4)	10	20.4(10.2-34.3)	5	19.2(6.6-39.4)
Tenderness	26	23.4(15.9-32.4)	24	25.8(17.3-35.9)	3	6.1(1.3-16.9)	3	11.5(2.4-30.2)	11	9.9(5.1-17.0)	17	18.3(11.0-27.6)	10	20.4(10.2-34.3)	3	11.5(2.4-30.2)
Itch	1	0.9(0.0-4.9)	1	1.1(0.0-5.8)	1	2.0(0.1-10.9)	0	0(0-13.2)	0	0(0-3.2)	3	3.2(0.7-9.1)	1	2.0(0.1-10.9)	0	0(0-13.2)
Redness	6	5.4(46.1-65.3)	7	7.5(3.1-14.9)	1	2.0(0.1-10.9)	0	0(0-13.2)	2	1.8(0.2-6.4)	4	4.3(1.2-10.6)	2	4.1(5.0-14.0)	0	0(0-13.2)
Total																
Systemic	62	55.9(46.1-65.3)	50	53.8(43.1-64.2)	8	16.3(7.3-29.7)	4	15.4(4.4-34.9)	18	16.2(9.9-24.4)	30	32.3(22.4-42.8)	18	36.7(23.4-51.7)	5	19.2(6.6-39.4)
Local	40	36.0(27.1-45.7)	37	39.8(29.8-50.5)	7	14.3(5.9-27.2)	4	15.4(4.4-34.9)	20	18.0(11.4-26.4)	29	31.1(22.0-41.6)	13	26.5(14.9-41.1)	6	23.1(9.0-44.0)
Overall	62	55.9(46.1-65.3)	60	64.5(53.9-74.8)	11	22.5(11.8-36.6)	5	19.2(6.6-39.4)	28	25.2(17.5-34.4)	35	37.6(27.8-48.3)	21	42.9(28.8-57.8)	7	26.9(11.6-47.8)
Medical attention	4	3.6(1.0-9.0)	24	25.8(17.3-35.3)	5	10.2(3.4-22.2)	1	3.9(0.1-19.6)	2	1.8(0.2-6.4)	3	3.2(0.7-9.1)	7	14.3(5.9-27.2)	2	7.7(0.9-25.1)

BNT: Comirnaty (BNT162b2, BioNTech-Pfizer, Mainz, Germany/New York, United States (US); ChAd: Vaxrevia (ChAdOx1/nCoV-19, AstraZeneca, Cambridge, United Kingdom (UK).

Please note individuals could report more than one reaction therefore totals do not match the sum of the symptoms.

S.6 Overall symptom severity by vaccination schedule

Severity of combined local and systemic reaction after prime and boost vaccination by vaccination schedule.

Severity is presented as the highest severity as self-reported by the participant. Grading was categorised as Grade 1 = Mild – easily tolerated with no limitation on normal activity, Grade 2 = Moderate – some limitation of daily activity, Grade 3 = Severe – unable to perform normal daily activity and Grade 4 = Emergency department or hospital admission required. Individual graphs are grouped into "No previous infection" - those that reported no previous symptoms nor a positive test, "Previous symptoms" – those that reported symptoms but no positive test, "Positive test" – have previously tested positive for SARS-CoV-2, "Under 50 year-olds", "Over 50 year-olds", "Females" and "Males".

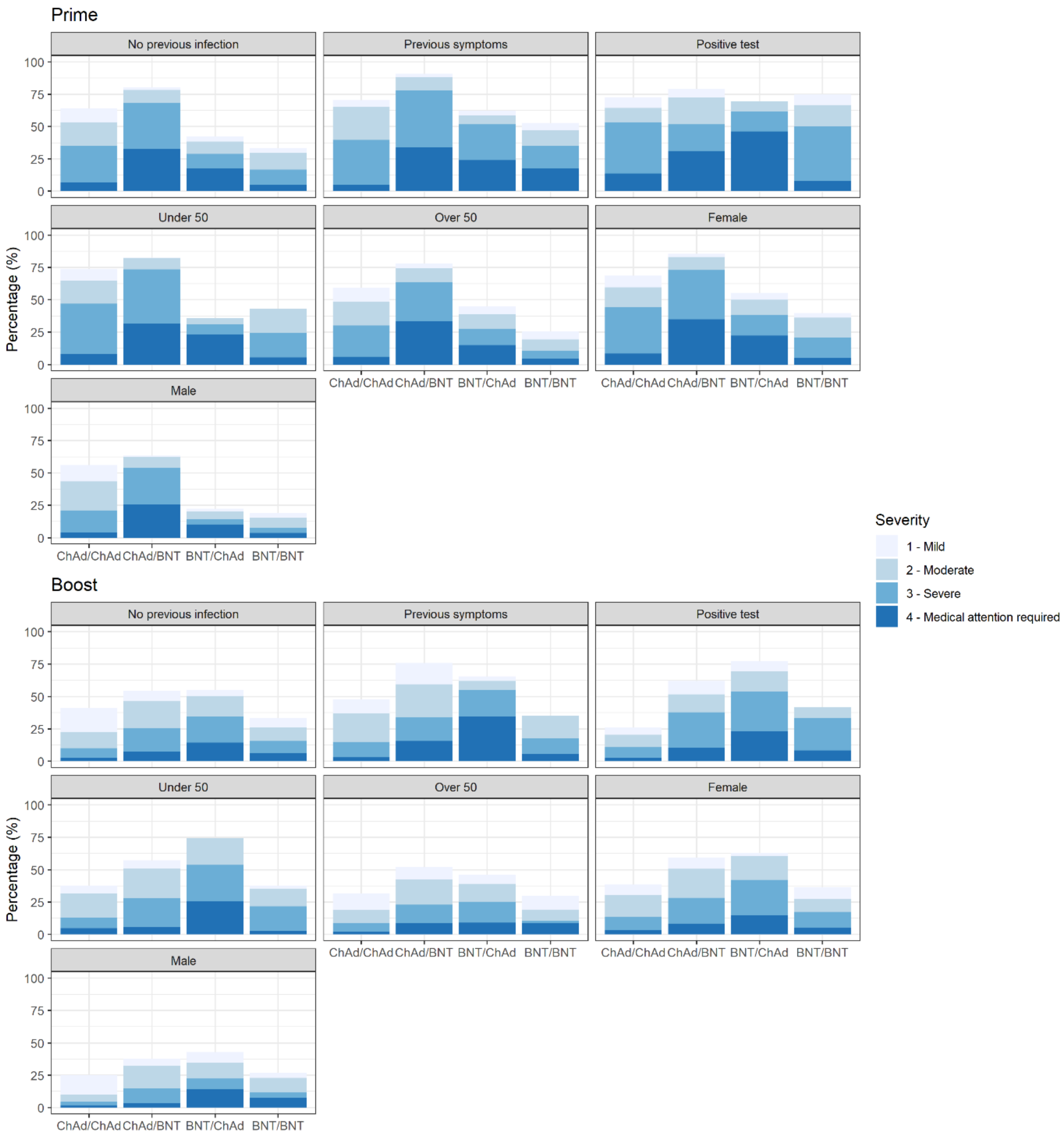

S.7 Severity of reactions after first and second dose by vaccination schedule

The total number of people who did not report previous symptoms of COVID-19 nor confirmed infection and reported severe reactions (Grades 3 or 4) for their second dose of the vaccine by those that reported severe reactions after their first dose and those that reported non-severe reactions (Grades 0-2) after their first dose by vaccination schedule.

Grade 0 = No symptoms, Grade 1 = Mild – easily tolerated with no limitation on normal activity, Grade 2 = Moderate – some limitation of daily activity, Grade 3 = Severe – unable to perform normal daily activity and Grade 4 = Emergency department or hospital admission required.

		Severe Reaction After 2nd dose	
Severe reaction after 1st dose	2nd dose	n/N	% (95%CI)
ChAd	ChAd	22/101	21.8 (14.2-31.1)
	BNT	79/267	29.6 (24.2-35.5)
BNT	ChAd	17/36	47.2 (40.4-64.5)
	BNT	6/14	42.9 (17.7-71.1)
Non-severe reaction after 1st dose			
ChAd	ChAd	8/186	4.3 (1.9-8.3)
	BNT	21/123	17.1 (10.9-24.9)
BNT	ChAd	26/89	29.1 (20.1-39.8)
	BNT	7/70	10.0 (4.1-19.5)

BNT: Comirnaty (BNT162b2, BioNTech-Pfizer, Mainz, Germany/New York, United States (US); ChAd: Vaxrevia (ChAdOx1/nCoV-19, AstraZeneca, Cambridge, United Kingdom (UK).
